# The coverage gap in PREVENT: Why cardiovascular risk prediction needs a lab-free complement

**DOI:** 10.1016/j.ajpc.2026.101782

**Published:** 2026-08-04

**Authors:** Rebecca Arden Harris, Warren B. Bilker, Douglas S. Jacoby, Michael G. Levin, Richard C. Wender, Sean Hennessy

**Affiliations:** a Department of Family Medicine and Community Health, Perelman School of Medicine, University of Pennsylvania, Philadelphia, PA, USA; b Penn Cardiovascular Institute, University of Pennsylvania, Philadelphia, PA, USA; c Leonard Davis Institute of Health Economics, University of Pennsylvania, Philadelphia, PA, USA; d Department of Biostatistics and Epidemiology, University of Pennsylvania, Philadelphia, PA, USA; e Division of Cardiovascular Medicine, Perelman School of Medicine, University of Pennsylvania, Philadelphia, PA, USA; f Center for Preventive Cardiology and Lipid Management, Perelman School of Medicine, University of Pennsylvania, Philadelphia, PA, USA; g Michael J. Crescenz VA Medical Center, Philadelphia, PA, USA; h Center for Public Health Initiatives, University of Pennsylvania, Philadelphia, PA, USA; i Center for Real-World Effectiveness and Safety of Therapeutics, Department of Biostatistics, Epidemiology and Informatics, Perelman School of Medicine, University of Pennsylvania, Philadelphia, PA, USA

**Keywords:** Cardiovascular risk prediction, PREVENT equations, Missing data, Health disparities, Risk prediction models

## Abstract

The American Heart Association's PREVENT equations are now embedded in three major AHA/ACC guidelines governing hypertension, dyslipidemia, and cardiovascular-kidney-metabolic syndrome, including a specific risk threshold that gates initiation of GLP-1-based therapy and SGLT2 inhibitors. This rapid guideline uptake has outpaced a persistent limitation: PREVENT requires laboratory values, chiefly lipids and estimated glomerular filtration rate, that are frequently missing in the populations where risk prediction matters most, including patients seen in community health centers, emergency departments, and substance use treatment programs. Real-world validation studies across multiple health systems show that PREVENT underestimates cardiovascular risk most severely in these same subgroups, with the largest underestimation among Medicaid-insured, uninsured, and Black patients. Imputation, whether simple substitution or multiple imputation, does not resolve this problem because the probability that a laboratory value is missing is itself associated with the underlying risk the model is trying to estimate, a form of informative missingness that standard imputation methods are not designed to address. We argue that the field should invest in developing and validating a standalone cardiovascular risk prediction model that does not require laboratory measures. Such a model would function as a complement to PREVENT, not a replacement, providing risk estimates for patients without laboratory data and helping identify those who warrant expedited evaluation. As PREVENT becomes the gatekeeper for guideline-directed therapy across three clinical domains simultaneously, closing this coverage gap is no longer a methodological refinement but a clinical implementation imperative.

## 1 Introduction

The American Heart Association's Predicting Risk of Cardiovascular Disease Events (PREVENT) equations represent a significant advance in cardiovascular risk prediction, incorporating kidney function and broadening the composite outcome to include heart failure [1]. Three major American Heart Association/American College of Cardiology (AHA/ACC) guidelines now endorse PREVENT: the 2025 Hypertension Guideline, which uses PREVENT for Stage 1 hypertension management [2]; the 2026 Dyslipidemia Guideline, which assigns PREVENT-ASCVD a Class I recommendation for adults aged 30–79 without established ASCVD [3]; and the 2026 CKM Syndrome Guideline, which uses a PREVENT-CVD score of ≥7.5% as a threshold for initiating GLP-1–based therapy or SGLT2 inhibitors in adults with type 2 diabetes and stages 2 or 3 CKM syndrome [4]. Yet real-world implementation has exposed two problems that guideline endorsement cannot fully resolve. First, PREVENT requires laboratory values that are frequently missing in the populations where accurate risk prediction matters most, leaving a substantial fraction of high-risk patients unscored. Second, calibration tends to degrade in disadvantaged subgroups even when missingness is addressed through imputation in the research design, because missingness is plausibly correlated with the cardiovascular risk the model is trying to estimate, a pattern known as “missing not at random” (MNAR) that standard imputation cannot correct [5,6]. Both problems are consistent with a common source: a structural mismatch between the derivation population, where laboratory predictors were complete by design, and the deployment population, where they are not. As PREVENT becomes embedded across three guideline domains simultaneously, patients for whom a score cannot be generated are excluded not only from quantitative risk estimation but from algorithm-driven pathways to guideline-directed therapy. The solution lies in developing alternative prediction models that do not require laboratory predictors in the first place. Such a model provides a risk estimate when current laboratory values are unavailable, complementing rather than replacing laboratory-based assessment.

## 2 The laboratory coverage problem

PREVENT’s base model requires lipid values and estimated glomerular filtration rate (eGFR), with optional add-on models incorporating urine albumin-to-creatinine ratio and hemoglobin A1c [1]. In the controlled environment of derivation, required predictors were complete by design. In PREVENT’s own derivation cohorts, hemoglobin A1c was missing in 100% of several major research cohorts [1]; in the largest multinational validation, albumin-to-creatinine ratio was missing in 84% of new North American cohorts [7]. In real-world practice, laboratory missingness is concentrated exactly where it is most consequential. Evidence from a national study indicates that among low-income adults in Medicaid non-expansion states, uninsured individuals were substantially less likely to receive indicated cholesterol monitoring than their insured counterparts (72.6%vs. 93.7%), suggesting that missing lipid measurements in EHR data may partly reflect differential access to preventive care rather than random missingness [8]. EHR-based validation studies confirm this. In one large academic health system validation, 52% of lipid values were missing and only 31% of eligible patients had complete data; those with complete laboratory data differed systematically from the imputed cohort by insurance status and Area Deprivation Index (ADI), with Medicaid-insured and highest-ADI patients showing the worst calibration [9]. Independent data from our own institution corroborate the pattern in a different clinical context: lipid values were absent in >90% of adults with documented stimulant use, consistent with broader failures of laboratory capture across care settings rather than a characteristic of this population alone [10]. The data missingness problem is most pronounced in community health centers, emergency departments, urgent care centers, and substance use treatment programs, settings where the laboratory values that PREVENT requires are rarely on hand, whether because the infrastructure is limited or because the encounter is not aimed at primary prevention.

Across multiple real-world validation studies, PREVENT has underestimated cardiovascular risk most severely in the subgroups with the greatest laboratory data gaps [11], including a 46% underestimation among Black adults aged 30–39 [12]. The largest multinational validation to date, spanning 6.4 million individuals, did not report calibration by race, insurance status, or socioeconomic disadvantage, leaving this equity-relevant question unaddressed at scale [7].

## 3 Why imputation alone is not enough

These findings are consistent with the hypothesis that calibration failures track with data quality and outcome ascertainment. The populations most burdened by miscalibration are also the most likely to go unscored because of missing laboratory data, a pattern consistent with MNAR. The problem begins upstream in the derivation data itself. The Optum Data Warehouse, which supplies >90% of PREVENT's derivation sample, relies on billing and EHR data in which ASCVD events may be underreported, particularly in disadvantaged subgroups where documentation gaps are most common [13]. Even if laboratory values were complete, differential outcome capture could still bias the baseline hazard downward in the populations where calibration has performed worst.

Multiple imputation depends on the missing-at-random assumption, which is violated when missingness is MNAR [14,6]. In practice, imputation operates at the cohort level rather than substituting for an individual patient's missing laboratory result at the point of care. Recalibration addresses a different problem: it may improve performance, but not coverage, because the initial risk estimate still requires laboratory values.

## 4 The case for a lab-free complement

We therefore propose that the field invest in developing and validating cardiovascular risk prediction models that do not require laboratory values, as a complement to PREVENT rather than a replacement [15]. Such models would apply to any patient with routinely available clinical data, including the non-laboratory predictors already embedded in PREVENT (age, sex, blood pressure, BMI, smoking status, history of diabetes, and medication use), without requiring lipid panels or renal function tests. Importantly, an indicator for laboratory availability could itself be included as a predictor, since the absence of testing is informative about healthcare access and cardiovascular risk. This would avoid restricting derivation to a selected subgroup while leveraging the possibility that laboratory missingness may carry independent information about healthcare utilization, care access, or underlying risk.

PREVENT included eGFR and lipids for good reasons. A meta-analysis showed that adding eGFR and albumin-to-creatinine ratio to traditional risk factors improved discrimination for heart failure and cardiovascular mortality [16]. Lipids capture atherogenic burden that BMI approximates only crudely: a patient with BMI 28 and LDL 190 mg/dL has a fundamentally different risk profile from one with BMI 28 and LDL 90 mg/dL. Lipids and kidney function retain real predictive value; the problem is that a risk equation cannot guide prevention for patients it cannot score. A further limitation deserves acknowledgment: in disadvantaged populations, undiagnosed dyslipidemia or impaired kidney function may lead a lab-free model to underestimate risk in some individuals who would be classified as high risk if laboratories were obtained. That is a real tradeoff. But the alternative for these patients is often no estimate at all. Whether a lab-based model would perform substantially better in these specific populations has not been demonstrated, and a well-specified lab-free model incorporating history of diabetes, antihypertensive use, statin use, and smoking status can capture substantial metabolic and atherogenic burden without direct lipid or eGFR measurement.

## 5 Tradeoffs and limitations

One might argue that the more direct solution is to ensure disadvantaged populations receive laboratory testing, which would simultaneously enable risk estimation, diagnosis of dyslipidemia and CKD, and treatment initiation. We agree that expanding laboratory access is essential and should proceed in parallel. Yet this has been the field's default strategy for decades, and the persistence of large-scale laboratory missingness suggests that access barriers remain unresolved. For many patients, obtaining laboratory testing requires additional appointments, transportation, time away from work, copays, and childcare arrangements. A lab-free model therefore serves as a primary risk estimation pathway when laboratory measurements are unavailable, often providing the only completed risk assessment within the clinical encounter for patients who cannot reliably access downstream testing. That said, a lab-free risk score does not by itself enable pharmacotherapy initiation for all indicated treatments; starting an ACE inhibitor without creatinine and potassium, for example, would be clinically inappropriate regardless of estimated risk. Its immediate clinical value lies in triage: identifying patients who warrant same-visit laboratory ordering, prioritized follow-up, or care coordination in settings where the prevention pathway would otherwise not begin, and supporting risk-informed counseling and referral decisions that are currently made without quantitative risk assessment. In this way, the model does not replace laboratory-based care pathways but helps determine which patients enter them.

## 6 A hybrid fallback, and the case for a standalone model

One alternative is to retain laboratory-based models while using a validated lab-free fallback for patients without current laboratory data. This approach mirrors conditional model use elsewhere in clinical prediction, such as Wells criteria followed by d-dimer testing. But if the fallback is derived and validated only among patients who happened to have laboratory values available, it inherits the same representativeness problem as PREVENT. A fallback derived in the full eligible population, including patients who currently go unscored, is a standalone lab-free model. That is what we propose, with calibration assessed both in the overall population and among patients missing at least one PREVENT-required laboratory value, the population the model is intended to serve.

Non-laboratory models have not been widely adopted in the United States, largely because they have been framed as tools for resource-limited settings rather than as solutions to data-availability problems affecting US clinical populations [17]. Existing lab-free tools illustrate this framing. The WHO non-laboratory risk charts were developed and validated explicitly for 21 global regions with variable laboratory access [18], and a non-laboratory score was recently validated against a laboratory-based score in a Brazilian community cohort, again outside a US clinical context [19]. No major US guideline has endorsed a lab-free approach for primary prevention. The expanding role of PREVENT in US guidelines makes this gap increasingly consequential. A guideline standard of care that cannot generate an estimate for a substantial proportion of EHR patients demands a complement, not just a workaround. Our group recently demonstrated feasibility by developing and internally validating a lab-free cardiovascular risk model among adults who use nonmedical stimulants, achieving good discrimination and calibration [10]. Although external validation is required before broader application, this work illustrates that clinically useful prediction models can be developed without laboratory inputs.

## 7 What would be required

What is needed is a rigorously developed, contemporary lab-free cardiovascular risk prediction model that meets four criteria. First, it should be derived from populations regardless of laboratory availability. Second, it should predict PREVENT's composite outcome using the same event definitions. Third, it should report both discrimination and calibration explicitly by race, insurance status, and neighborhood deprivation, as well as separately among individuals missing at least one PREVENT-required laboratory value, since this is the population the model is intended to serve. Fourth, it should be embedded as the clinical default when laboratory values are absent.

Such a model could be derived from national EHR repositories, including the All of Us Research Program and other multi-system consortia, with prespecified validation in systems stratified by payer mix and ADI distribution and explicit benchmarking of subgroup-specific performance against PREVENT's observed results. Implementation would require AHA/ACC endorsement of a lab-free complement to PREVENT, along with integration into clinical decision support that automatically defaults to the lab-free calculator when laboratory values are absent, mirroring PREVENT's own add-on architecture for optional predictors and extending the same logic in the opposite direction. Calculator uptake has historically been limited by workflow burden and uneven integration into routine practice [20,21]; automatic EHR-based defaulting, rather than clinician-initiated lookup, would be essential to avoiding the same fate.

## 8 Conclusion

With three major AHA/ACC guidelines now embedding the PREVENT equations, patients for whom a score cannot be generated are excluded not only from risk estimation but from the risk-threshold step of algorithm-driven pathways to guideline-directed therapy. Ensuring that risk estimation remains available for all patients regardless of laboratory data is no longer a methodological concern. It is a clinical imperative.

## Funding

RAH is supported by the 10.13039/100000026National Institute on Drug Abuse under award K23DA054157. MGL is supported by U.S. Department of Veterans Affairs Biomedical Research and Development Award IK2-BX006551; has received research grants from MyOme; and served as a consultant for BridgeBio, unrelated to the current work. This work does not represent the views of the Department of Veterans Affairs or the U.S. government.

## Disclosures

None

## CRediT authorship contribution statement

**Rebecca Arden Harris:** Conceptualization, Writing – original draft, Writing – review & editing. **Warren B. Bilker:** Writing – review & editing. **Douglas S. Jacoby:** Writing – review & editing. **Michael G. Levin:** Writing – review & editing. **Richard C. Wender:** Writing – review & editing. **Sean Hennessy:** Conceptualization, Writing – review & editing.

## Declaration of competing interest

The authors declare that they have no known competing financial interests or personal relationships that could have appeared to influence the work reported in this paper.
